# Endocrine and metabolic response to trauma in hypovolemic patients treated at a trauma center in Brazil

**DOI:** 10.1186/1749-7922-3-28

**Published:** 2008-10-06

**Authors:** Luiz CV Bahten, Fernando HO Mauro, Maria F Domingos, Paula H Scheffer, Bruno H Pagnoncelli, Marco AR Wille

**Affiliations:** 1 General surgery department, Cajuru University Hospital, Curitiba, Brazil

## Abstract

**Background:**

The metabolic changes in trauma patients with shock contribute directly to the survival of the patient. To understand these changes better, we made a rigorous analysis of the variations in the main examinations requested for seriously polytraumatized patients.

**Methods:**

Prospective analysis of patients with blunt or penetrating trauma with hypovolemic shock, with systolic arterial pressure (SAP) equal to or lower than 90 mmHg at any time during initial treatment in the emergency room and aged between 14 and 60 years old. The following exams were analyzed: sodium, potassium, blood test, glycemia and arterial gasometry. The tests were carried out at intervals: T0 (the first exam, collected on admission) and followed by T24 (24 hours after admission), T48 (48 hours after admission), T72 (72 hours after admission).

**Results:**

The test evaluations showed that there was a tendency towards hyperglycemia, which was more evident upon admission to hospital. The sodium in all the patients was found to be normal upon admission, with a later decline. However, no patient had significant hyponatremia; there was no significant variation in the potassium variable; the gasometry, low pH, BE (base excess) and bicarbonate levels when the first sample was collected and increased later with PO_2 _and PCO_2 _showing only slight variations, which meant an acidotic state during the hemorrhagic shock followed by a response from the organism to reestablish the equilibrium, retaining bicarbonate. The red blood count, shown by the GB (globular volume) and HB (hemoglobin) was normal upon entry but later it dropped steadily until it fell below normal; the white blood count (leukocytes, neutrophils and band neutrophil) remained high from the first moment of evaluation.

**Conclusion:**

In this study we demonstrated the main alterations that took place in patients with serious trauma, emphasizing that even commonly requested laboratory tests can help to estimate metabolic alterations. Suitable treatment for polytraumatized patients with hypovolemic shock is a challenge for the surgeon, who must be alert to endocrinal and metabolic changes in his patients. Based on these alterations, the surgeon can intervene earlier and make every effort to achieve a successful clinical result.

## Background

The endocrinal and metabolic alterations in the trauma maintain vascular homeostatis in addition to hydroelectrolitical, nutritional and hormonal integrity. Generally, this complex response is harmonic and ordered, resulting in the restoration of homeostasis. When this response is excessive, there will be profound imbalance in the homeostasis, resulting in these metabolic changes continuing into shock and the metabolic blockage of several organs, delaying the improvement of the patient or resulting in death.

The best knowledge of definitive treatment for traumatic lesions and endocrinal and metabolic alterations to trauma can be useful in conducting planning and adaptation, improving treatment of patients, reducing their recovery time and improving prognosis.

In this study, we aimed to identify early metabolic and hydroelectrolitical alterations resulting from serious trauma through simple, routine laboratory tests which are available even at small hospitals.

This study was conducted at the Cajuru University Hospital (CUH), run by the PUCPR University in Curitiba, the capital city of Paraná State in the south of Brazil. The CUH is a respected center for traumas and emergencies in the south of the country and has 300 beds, of which twenty are in the intensive care unit.

## Methods

Following approval by the committee in charge of ethics in research at the institution where the study was to take place, the results of the laboratory exams of the patients who met the criteria for inclusion in the study were consecutively analyzed. The collection of data was begun in the second half of 2007 and finalized in the first semester of 2008.

Fifty-one patients of both sexes, aged 14–60, suffering from moderate to serious trauma with systolic arterial pressure ≤90 mmHg at any time during admission were included in the study. Following analysis of the laboratory tests, the metabolic responses at the different assessment times were compared.

For each of the variables that were normal, the null hypothesis that the averages were the same at the four evaluations was tested, versus the alternative hypothesis that in at least one instance there was a different average from the rest. In the tables below, the values of averages and standard deviations are given and the p values of the statistical tests. We highlight only the results that showed significant variations throughout the study.

### 1. Study Model

The results of the study were given by averages and standard deviations or medians, minimum values and maximum values. To compare the evaluations with the variables that met the condition of normalcy, the variance analysis with repeated measurements was used and the LSD multiple comparison test. For variables that did not meet the conditions of normalcy, this comparison was made by using Friedman's non-parametric test and the Wilcoxon non-parametric test for multiple comparisons. Values of p < 0.05 indicated statistical significance. For multiple comparisons using the Wilcoxon test, the level of significance was corrected by Bonferroni. In this case, values of p < 0.008 indicated statistical significance.

### 2. Methods

The prospective analysis was carried out in patients with blunt or penetrating trauma with hypovolemic shock, evaluated by SAP equal to or lower than 90 mmHg at any time during initial treatment in the emergency room and aged between 14 and 60. The following exams were analyzed: sodium, potassium, blood test, glucose, glycemia and arterial gasometry. The test were carried out at intervals: T0 (the first exam, collected on admission) and followed by T24 (24 hours after admission), T48 (48 hours after admission), and T72 (72 hours after admission).

## Results

Our study showed that relevant alterations were observed in the laboratory tests that were requested when compared with the values considered normal by our laboratory.

**Figure 1:**
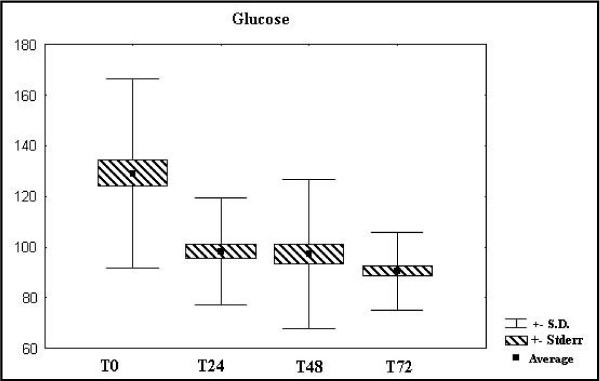
Glucose variation.

Hyperglycemia was found in most of the patients upon admission to the hospital, with a later tendency to normalize, although the glycemic levels remained high even during fasting. (Figure 1: glucose variation)

**Figure 2:**
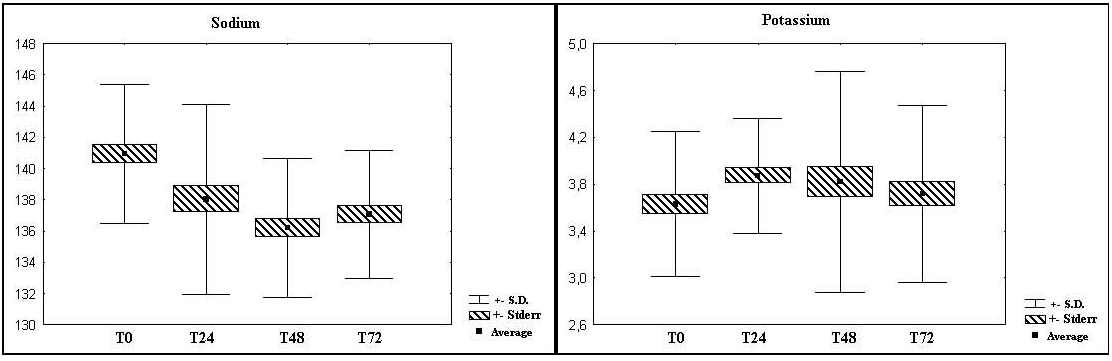
**Variation of electrolytes, sodium and potassium.** Normal values: sodium (137–145 mmol/L) and potassium (3,5–5,1 mmol/L).

The sodium values in all patients were found to be normal upon admission, with a later decline. However, no patient had significant hyponatremia, although the reduced sodium plasma levels had already been detected after twelve hours. Nevertheless, there was no statistical difference in the potassium analysis during the 72 hours. (Figure 2: variation of electrolytes, sodium and potassium)

All the patients involved in this study were submitted to a minimum infusion of 2000 ml of crystalloid solution in the initial approach. The criteria for transfusion of red blood cell concentrate was a lack of immediate response to an infusion of up to 4000 ml of crystalloid and levels of Hemoglobin that were < 7 mg/dl at any time during hospitalization. Out of a total of 51 patients, 16 met these criteria.

**Figure 3:**
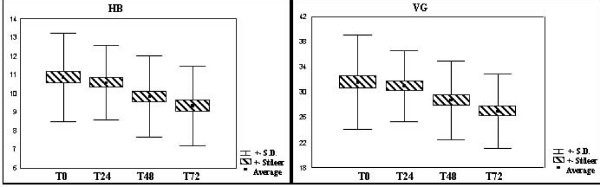
**Variation of red series.** Normal values: HB (12,5–16,5 g/dL) and VG (37,5–49,5%).

The red blood count, represented in this study by the variables Globular Volume (GV) and Hemoglobin (HB), was found to be normal upon entry but steadily reduced until it fell below normal. (Figure 3: variation of red series)

**Figure 4:**
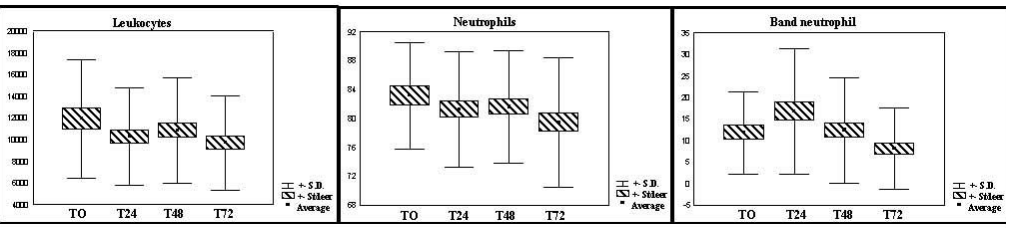
**Variation of white series.** Normal values: Leukocytes (3500–10000/mm^3^), neutrophils (45–70%), band neutrophil (until 8%).

In the white blood count the leukocytes, neutrophils and basophils were analyzed. The values remained high from the first evaluation, demonstrating the prompt response of the organism to tissue injury. (Figure 4: variation of white series)

**Figure 5:**
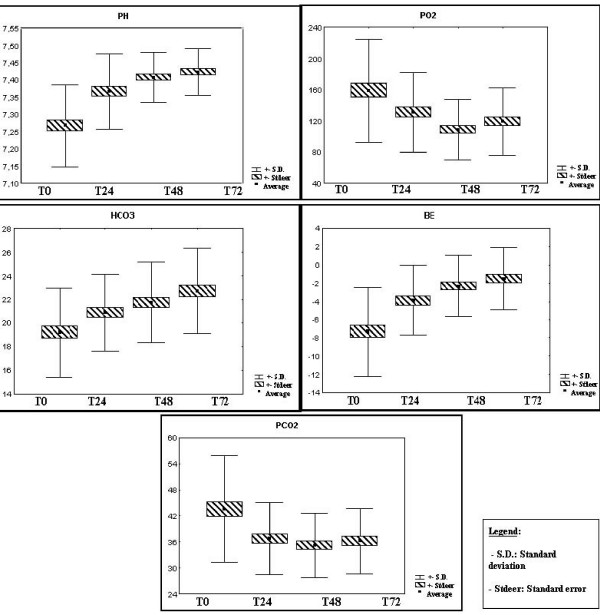
**Gasometry variation. **Normal values: pH (7,35–7,45), PCO_2 _(35–46 mmHg), PO_2 _(75–100 mmHg), HCO_3 _(20–30 mmol/L), and BE (-2 – +2).

The gasometry variables that were analyzed were bicarbonate (HCO_3_), PO_2 _(oxygen pressure), PCO_2 _(carbon dioxide pressure), BE (base excess) and pH, as these are more important when evaluating patients in serious condition. (Figure 5: gasometry variation)

## Discussion

Trauma continues to be the main cause of death in patients under forty-five in industrialized and developed countries. There are three different types of death by trauma. The first is immediate death, or sudden death, which happens on the spot and the main causes of which are injuries such as ruptured aorta, cerebral stem lacerations or large amputations. The second is early death, occurring early in the "golden hour" of the trauma and resulting from compromised airways, as in the hypertensive pneumothorax or hemorrhagic shock by intra-abdominal or intra-thorax hemorrhage or rupture of the pelvic ring or even serious traumatic cerebral lesions like cerebral edema or intra-cranial bruises. The third is deaths which occur days or weeks after the trauma, the main causes being septic shock and multiple organ failure.[1]

Death by sepsis or trauma occurs due to acute alterations resulting from circulatory collapse, firstly, complex pulmonary failure, secondly, and, lastly, if the first two mechanisms of death have been avoided, multiple organ and systems failure[2].

With serious traumatism, the stimuli are many and intensified, and the reflexes are aimed at an integrated attempt by the organism to restore cardiovascular stability, preserve the oxygen supply, mobilize the caloric substrates and increase the supply of fundamental substrates, especially glucose.[3]

If the trauma victim survives, profound and systemic modifications occur which affect mainly the organic metabolism, ensuring the supply of energetic substrates and raw materials for the reconstruction of damaged tissues[4].

To understand better the response of the organism to trauma, it was necessary to divide it into two different phases. The first, or ebb, phase lasts from two to three days and is characterized by frank hemodynamic instability, here represented by hypotension, hypovolemia, reduced blood flow, increased systemic vascular resistance, catecholamines, glucocorticoids and mineralocosticoids, disturbances in the transport of oxygen beyond increased intake and depletion of hepatic glycogen.[3,5,6].

Following the initial injury, the flow phase, characterized by hyperdynamism in response to the aggression suffered by the organism, represented by water retention, increased vascular permeability and reduced vascular resistance with growing levels of glucocorticoids and catecholamines, resulting in hyperglycemia and proteolysis.[6] However, in some patients there is imbalance of these compensatory metabolisms, which organic stress does not resolve it and there is systemic dysfunction, which may or may not be linked to the infection[3,5]. There are alterations to the state of consciousness, metabolic acidosis, peripheral intolerance to glucose, fever, tachypnea, leukocytosis, hypoxemia, hypocapnia, hyperbilirubinemia and increased urinary and plasmatic creatinine[6].

The total body metabolic response is the combined response of the organs and their individual metabolic demands as modulated by the neuroendocrine system and exogenous nutritional support. In general, the onset of "trauma disease" is associated with bedrest of the muscles of ambulation concurrently with increased work of the cardiac and respiratory muscles and an increased demand for plasma proteins, red cells, wound healing, and activity of the systemic antibacterial systems. The neuroendocrine system may be divided into its catabolic and anabolic components[2].

In this context, improved treatment of patients in emergency services aims to reduce trauma-related deaths. It is important to understand the metabolism of critical patients, evaluating the main physiological parameters of the patient in terms of metabolic stress, which upsets the restoration of his homeostasis. It should not be forgotten that when dealing with a trauma victim, every response will be governed by the cellular catabolism[2].

The hyperglycemia found in the trauma, induced by the catecholamines, seems to be the result both of increased hepatic release of glucose and reduction in glucose clearance.[7] The increase in plasmatic glucose is proportional to the gravity of the wound, which is reflected in a positive correlation between the gravity of the wound and the concentration of glucose in trauma victims. The presence of hyperglycemia gives the brain a source of calories that can be readily used and may be important to initial survival. Alterations to the metabolism of carbohydrates which takes place during trauma include an increase in the hepatic production of glucose, mediated by the glucagons (hepatic glycogenesis) and a disturbance in peripheral glucose capture[3], in response to the reduced insulin levels, a classic anabolic hormone and increase in counter-insulin hormones, now catabolic, such as glucagons, cortisol and epinephrine[9]. In our study, hyperglycemia was found in most of the patients upon admission to the hospital, with a later tendency to normalize, although the glycemic levels remained high even during fasting.

The degree of disturbance in the hydrolectrolitical balance after a lesion depends, in part, on functional extracellular volume that has been lost from the capacity to respond on the part of the neuroendocrinal, renal and circulatory systems, the gravity of the lesion, the quality and quantity of administered liquid, the age of the patient, pre-existing diseases, concomitant medications and the anesthetic agents that are used[3].

The main cause of the hypovolemic hyponatremia found in this type of patient is a hemorrhage. In critical patients, the depletion of sodium and the reduced vascular volume, upon arrival of hypovolemia, may be an iatrogenic consequence of renal losses, unleashed by the metabolic stress suffered by the organism[5].

The sodium is significantly altered in trauma patients with shock. We found a great variation after the first hours of treatment, mostly due to initial handling with the infusion of crystalloid solutions (Ringer Lactate and Sodium Chlorate 0.9%) and later the Na-K-ATPase pump[8]. Initially the first response to a hypovolemic injury is the depletion of sodium by the considerable removal of liquid without compensation. Hours after the trauma, the organism releases mediators capable of restoring the patient's volemia with the release of ADH and aldosterone, along with the infusion of crystalloids, the purpose of which is also to maintain the hyrdroelectrolitic balance[5,9]. We attribute the sodium alterations to the vigorous volemic replacement to which patients were submitted.

The increased sodium in the cell stimulates enzymatic activity of the Na+, K+-ATPase, which pumps the sodium out of the cell in order to restore the potential of the cellular membrane. Depolarization is a vital and essential activity for the functioning of the neurons and myocardial cells. In shock patients, the activity of the Na+, K+-ATPase is hindered because the anaerobic aggression of the shock impedes the arrival of sufficient energy to the cells which is necessary to maintain the active transport of the sodium ions by the Na+, K+-ATPase. Initially, there could be increased release of this ion by the muscular lesion and then a reduction would be expected due to the release of aldosterone.

Potassium is the main intracellular cation. In cases of metabolic injury, the organism responds with the diffusion of potassium out of the cell. What is responsible for this phenomenon is aldosterone, released by angiotesin II and the increase of potassium in the serum, resulting from the hypovolemia in these patients. The role of the aldosterone here is to act in the exchange of intraluminar sodium for intracellular K^+^. As the plasmatic level of adosterone increases, a great deal of Na^+ ^is reabsorbed and more K^+ ^is excreted[5]. Due to potassium being the most abundant intra-cellular ion, there could initially be an increase through the release of this ion by the muscular lesion and then a drop would be expected because of the release of aldosterone. However, there was no statistical difference in the analysis of potassium over the 72 hours. In this study, there was a tendency to hyponatremia from twelve hours after trauma[5,9]

The magnitude of the physiological aggression suffered by the organism is directly proportional to the volume of lost blood. Two responses may be seen in these patients: if blood loss is low (<15% of blood volume), the organism responds with a vasoconstriction; if blood loss is high (15–40% of blood volume), the response is hypotension, with a hemorrhage and imminent risk of life[10].

Hypovolemic shock, a type of hypodynamic shock, is related to the low cardiac output and increased systemic vascular resistance (vasoconstriction). The massive drop in red blood cells, hemoglobin and hematrocrit are the main laboratory markers of hypovolemia, excluding the first period after hemorrhage has begun. Clinically, compromised cardiac output is by primary reduction of the venous return. If cardiac output falls, the arterial pressure will follow suit, affecting organic perfusion[10]. However, the immediate stimulus of the baroreceptors, located in the carotids, atria and ventricles leads to neuro-humoral hyperactivation. Thus the statement that the hemorrhage is accompanied by a global compensation orchestrated by the sympathetic nervous system and aided by most of the systems of organs in the body[11]. The catecholamines increase the synaptic clefts of the blood vessels and heart, especially noradrenaline and adrenaline in the circulation itself, resulting in an increase in frequency and contractility, resulting in arteriolar and venous vasoconstriction. The global response to this sequence of alterations is increased systemic vascular resistance in order to avoid a drop in arterial pressure, increased cardiac output, resulting form the direct effect of the catecholamines and increased venous return (vasoconstriction)[9]. The red blood count was found to be normal upon entry but steadily reduced until it fell below normal. This alteration was expected as all the patients had serious hypovolemic shock and were given a large infusion of crystalloids and blood derivatives. Nevertheless, the values were sharply altered since the organism cannot control the sudden loss of blood immediately. The variations found in the responses of patients to trauma and hemorrhagic shock showed, after the evaluation of the red blood count, a significant drop in hemoglobin and by a relatively smaller correlation of red blood cells and a less significant drop in globular volume.

Other factors related to shock stimulate macrophages to produce and release a cytokine, tumor necrosis factor alpha (TNF-alpha), one of the important inflammatory mediators of shock. This cytokine stimulates the invasion of leukocytes to the tissues by increasing the formation of neutrophils in the bone marrow (leukocytosis) and, at the same time promoting the endothelial expression of leukocyte adhesion molecules. The neutrophils may be responsible for the tissue lesion which, when activated, release oxygen free radicals, N-chloramines and proteolytic enzymes.[2,5,9]. In our study, the white blood analysis, leukocytes and their precursors, revealed leukocytosis, mainly at the cost of the neutrophils, as expected, and the band neutrophil, unleashing an important response in the maintenance and exacerbation of the inflammatory process, not necessarily meaning an ongoing infectious process.

The basic acid alteration predominant in shock and metabolic acidosis resulting from insufficient supply of tissues with sufficient blood flow and oxygen, which are highly necessary to the mitochondrial aerobic metabolism. Hemorrhagic shock mainly consists of hypovolemia, with insufficient cardiac filling pressure, hemodilution, of the hemoglobin and reduced cardiac output, which in fusion, reduce the capacity to carry oxygen per unit of blood volume[5,13].

There is no shock without alterations to the cellular metabolism. The explanation for acidosis in shock patients is the incapacity to recapture the H^+ ^released when the ATP is oxidized ADP. The ADP is not converted into ATP because of mitochondrial insufficiency to carry out oxidative phosphorylation[5]. With a low oxygen supply, the organism ceases mitochondrial metabolism via the Krebs cycle, impeding oxidative phosphorylation and ceasing to produce ATP and CO_2_, and beginning to produce pyruvate via glycolysis, which accumulates in the cytoplasm until it is converted into lactate and then it is released[5,9]. Thus, there is now lactic, the main parameter of oxidative insufficiency of the cell. The alterations of the physiological metabolic pathways leads to the development of metabolic acidosis with hyperlactatemia[13].

But the fall in tissue perfusion caused by the hypovolemia and cellular hypoxia is also seen through other parameters evaluated in the arterial gasometry. Not only do patients with metabolic injury develop metabolic acidosis, but they also react with other compensatory mechanisms to this cellular stress in order to maintain the HCO_3_/CO_2 _relationship, (buffering system) as close as possible to normal. Thus, the organism reduces the PCO_2 _through compensatory hyperventilation. We then find the answer to why the HCO_3 _and PCO_2 _are low. Even with these mechanisms, we can never say that this response will be complete because there is no pH correction to a normal level. Another interesting piece of information in the arterial gasometry that leads us to consolidate the initial idea of metabolic acidosis and later compensatory response is the base excess values. Negative values in an acute injury mean that there has been a reduction in the total number of bases, i.e., that the organism has lost bases resulting from a primary metabolic disturbance, in this case, metabolic acidosis.[2,3,5,9,14,15].

In serious trauma, there is a considerable fall in tissue perfusion, which impedes the arrival of nutrients and O_2 _to the cells and the purification of components derived from the metabolism such as CO_2 _and toxins. The accumulation of lactate (anaerobic metabolism), CO_2 _and other substances, leads to a progressive reduction in the pH with the consumption of bicarbonate. Almost immediately, chemo receptors found in the aortic arch modulate impulses afferent to the central bulbar, leading to hyperventilation (Kussmaul breathing) in their attempt to eliminate CO_2 _and reduce the acidemia levels. Nevertheless, these mechanisms cannot keep the pH normal and we see that initially this reaches close to 7.5. It is important to point out that a large number of the patients analyzed were submitted to invasive mechanical ventilation in order to carry out their surgical procedures. Based on the data given above, we see the development of mixed acidosis, with a predominantly metabolic component, mainly at the cost of the bicarbonate ion.

Although gasometry has good parameters for identifying criteria of the gravity and clinical condition of patients, it should not be used as a substitute for other tests such as lactate testing, considered as a more sensitive marker for the diagnosis of hypoperfusion and prognosis in patients suffering from hypovolemic shock.[16].

## Conclusion

In this study we demonstrated the main alterations in serious trauma patients, emphasizing that even commonly requested laboratory tests can estimate metabolic alterations. Suitable treatment for polytraumatized patients with hypovolemic shock is a challenge for the surgeon, who must be alert to endocrinal and metabolic changes in his patients. Based on these alterations, the surgeon can intervene earlier and make every effort to achieving a successful clinical result.

## Competing interests

The authors declare that they have no competing interests.

## Authors' contributions

The authors contributed equally to this work.
